# Sir Benjamin Ward Richardson

**Published:** 1896-12

**Authors:** 


Sir Benjamin Ward Richardson, Bart., M. D., F. R. S., etc., died of apoplexy at London, November 21, 1896, aged 68 years. He was honorary physician to the royal literary fund, the newspaper press fund and the national society of schoolmasters, at London. He was born in 1828, and was an honorary member of the Philosophical Society of America. He has been president of the Medical Society of London and was thirty-two times president of the St. Andrew's Medical Graduates' Association. He took an active interest in the development of bicycling and was president of the Society of Cyclists. He was knighted in 1893. He was a frequent contributor to medical literature, and was noted as an author, especially in hygiene.

